# Comparative Study on the Spreading Behavior of Oil Droplets over Teflon Substrates in Different Media Environments

**DOI:** 10.3390/polym14142828

**Published:** 2022-07-12

**Authors:** Junchao Wang, Yijun Cao, Guosheng Li

**Affiliations:** 1 Chinese National Engineering Research Center of Coal Preparation and Purification, China University of Mining and Technology, Xuzhou 221116, China; wjccumt@163.com; 2 School of Chemical Engineering and Technology, China University of Mining and Technology, Xuzhou 221116, China; 3 School of Chemical Engineering and Technology, Zhengzhou University, Zhengzhou 450001, China; lgscumt@163.com

**Keywords:** spreading behavior, oil droplets, Teflon, three-phase contact line (TPCL), dynamic contact angle, spreading mechanism

## Abstract

This paper comparatively investigated the spreading process of an oil droplet on the surface of highly hydrophobic solid (Teflon) in air and water media using a high-speed imaging technology, and analyzed their differences in spreading behavior from the perspective of empirical relations and energy conservation. Furthermore, the classical HD and MKT wetting models were applied to describe the oil droplet spreading dynamics to reveal the spreading mechanism of oil droplets on the Teflon in different media environments. Results showed that the entire spreading process of oil droplets on Teflon in air could be separated into three stages: the early linear fast spreading stage following $\theta \left(t\right)=\theta_{0}+kt$ , the intermediate exponential slow spreading stage obeying $\theta \left(t\right)=bt^{-3\alpha }$, and the late spreading stage described by $\theta \left(t\right)=\theta_{\mathrm{eq}}+a\times \exp\left(-t/T\right)$. However, the dynamics behavior of dynamic contact angle during the oil droplet spreading on Teflon in water could be well described by these expressions, $\theta \left(t\right)=\theta_{0}+kt$ and $\theta \left(t\right)=\theta_{\mathrm{eq}}+a\times \exp\left(-t/T\right)$. Clearly, a significant difference in the oil droplet spreading behavior in air and water media was found, and the absence of the intermediate exponential spreading stage in the oil–water–Teflon system could be attributed to the difference in the dissipated energy of the system because the dissipation energy in the oil–water–solid system included not only the viscous dissipation energy of the boundary layer of oil droplet, but also that of the surrounding water which was not included in the dissipation energy of the oil–air–solid system. Moreover, the quantitative analysis of wetting models suggested that the MKT model could reasonably describe the late spreading dynamics of oil droplets (low TPCL velocities), while the HD model may be more suitable for describing the oil droplet spreading dynamics at the early and intermediate spreading stages (high TPCL velocities).

## 1. Introduction

The dynamics of droplet impact play an important role in numerous natural processes [1,2,3] and practical applications such as industry [4,5,6,7,8,9], agriculture [10,11,12], and medicine [13,14,15]. The possible outcomes of a droplet impact on a solid surface include spreading, splashing, bouncing off, and sticking [16,17,18]. Obviously, the dynamics behavior of droplets impacting on solid surfaces is quite complex, and mainly depends on the surface characteristics of the solid and the properties of the fluid. If the droplet is a solution droplet containing surfactants, its dynamics also should be related to the physicochemical properties of the solution and the interaction between the solution and the solid surface. In this study, we focus on the spreading behavior of oil droplets on highly hydrophobic solid surfaces in different media environments with approximately negligible initial kinetic energy.

Droplet spreading is a complex multiphase flow process involving hydrodynamics and interface science, which is mainly controlled by three kinds of forces: inertial force, capillary force and viscous force. These forces are dependent on factors associated with the droplet parameters (size, density, viscosity, surface tension, impact velocity) and the solid surface characteristics (roughness and chemical property) [19,20]. Over the past few decades, scholars have conducted extensive research on the droplet spreading behavior from an experimental point of view. For example, Wang et al. [21] experimentally investigated the spreading dynamics of completely and partially wetted power-law fluids on solid substrates. They found that for the completely wetted system driven by the interaction of capillary force, disjoining pressure and viscous resistance near the three-phase contact line (TPCL), the evolution of the instantaneous spreading radius (R(t)) with time (t) could be described by the Tanner law [22], $R\left(t\right)=At^{\alpha }$; however, this power law was invalid for the partially wetted system. Therefore, they proposed a reasonable exponential power law, $R\left(t\right)=R_{\mathrm{eq}}\left[1-\exp\left(-at^{m}/R_{\mathrm{eq}}\right)\right]$, to describe the case of partial wetting, where $R_{\mathrm{eq}}$ is the equilibrium spreading radius, a is a coefficient related to the properties of the system and m is a fitting parameter that can reflect the spreading rate. Subsequently, they analyzed theoretically the spreading dynamics of Newtonian and non-Newtonian power-law fluids in the capillary spreading regime, and derived a new model to describe the spreading behavior of shear-thinning, shear-thickening, and Newtonian fluids in completely or partially wetted systems based on the classical energy-based approach [23]. Starov et al. [24] and Lee et al. [25] considered the spreading behavior of surfactant solution droplets over various substrates from both theoretical and experimental points of view, and suggested that the transfer of surfactant molecules from the water droplet onto the hydrophobic surface changed the wetting characteristics in front of the droplet on the TPCL. Shi et al. [26] performed an interesting experiment to study the spreading behavior of a conducted droplet on two glass plates coated with a conductive indium tin oxide (ITO) thin film within several milliseconds, and found that the spreading process in this time scale could be separated into two stages: a fast-inertial stage and a subsequent slow-viscous stage.

To reveal the mechanism of droplet spreading, scholars have proposed various theoretical models based on different considerations and assumptions. Among them, there are two classical models to describe the TPCL motion during spreading: the hydrodynamic (HD) model [27,28] and the molecular–kinetic theory (MKT) model [29]. The HD model considers the viscous dissipation of the liquid by applying the lubrication approximation on the Navier–Stokes equations, and assumes that the bulk viscous friction is the main resistance force for the TPCL movement [30]. The MKT model describes the TPCL movement based on the statistical kinetics of the individual molecular displacements occurring within the TPCL region by considering the interaction between the liquid and the solid. This model excludes the bulk viscous dissipation and assumes that the energy dissipation occurs only at the moving TPCL following the adsorption and desorption process. Although scholars also consider the viscosity in the modified MKT model [31,32], the viscosity only changes the displacement frequency. Therefore, the viscous effects have little influence in the MKT model. Despite the fact that the two models have been confirmed in some specific experimental systems and can explain the dynamics behavior of TPCL, in many cases, they cannot describe the experimental data of the entire TPCL velocity range alone [33,34]. As a result, a combined molecular–hydrodynamic model [35] has been proposed, which assumes that the energy dissipation is composed of viscous dissipation in the bulk liquid and the solid–liquid friction dissipation in the vicinity of TPCL. Apart from the theoretical progress, there are many numerical simulations on droplet spreading [36,37].

In summary, most studies on droplet spreading have focused on the spreading dynamics of pure liquids, surfactant solutions and/or their mixture solutions on various surfaces in air; however, the spreading behavior of oil droplets on solid surfaces in aqueous environments has been less studied. Recently, Agarwal et al. [38] analyzed the wetting, movement, and coalescence processes of underwater isooctane droplets on the surface of fiber substrates with different surface energies and surface roughness, and proposed that the hydrophobic–oleophilic surface was conducive to the efficient separation of oil and water. Han et al. [39] and Lü et al. [40] reported the spreading behavior of micrometer-scale oil droplets on different wettability surfaces in water systems using high-speed microscopic imaging technology, and established theoretical models that could describe the spreading behavior of underwater oil droplets on solid surfaces based on the energy conservation of the system before and after the rising oil droplet impacts the solid. Obviously, these works generally cover the rising process of oil droplets, the drainage process of the water film between oil droplets and solid surfaces, and the stabilization process when studying the spreading behavior of oil droplets on solids in the water environment, resulting in insufficient attention to the dynamic behavior of oil droplet spreading. Moreover, there are very few studies on the spreading behavior of oil droplets on the same solid surface in different media environments. To reveal the differences in the spreading behavior of oil droplets on solid surfaces in different media and to gain a deeper understanding of the spreading mechanism of oil droplets, we comparably studied the spreading behavior of millimeter-scale oil (oleic acid) droplets on the smooth hydrophobic solid (Teflon) surface in air and deionized water from the perspective of empirical relations and energy conservation, and the classical HD and MKT wetting models were applied to describe quantitatively the oil droplet spreading dynamics.

## 2. Materials and Methods

### 2.1. Materials

Oleic acid (purity > 99%, AR), a water-insoluble non-Newtonian fluid, was provided by Sinopharm Chemical Reagent Co., Ltd. (Shanghai, China). Teflon plates were purchased from http://www.tedpella.com (accessed on 20 May 2022) providing the microscopy products for science and industry, and cut to a size of 4 cm × 4 cm × 2 cm for spreading experiments. Their morphology was evaluated using an atom force microscope (AFM), and the root mean square (RMS) roughness values (<1.0 nm) were small enough that the influence of surface roughness on the oil droplet spreading behavior could be neglected. Besides, their surface wettability was also characterized by the sessile drop method with a DSA100 (KRÜSS, Hamburg, Germany), and the average static water contact angle was 117.1 ± 2.15°. It is worth noting that these Teflon plates must be thoroughly cleaned and dried before the spreading experiment, and the cleaning method can be seen in [41].

### 2.2. Experimental Apparatus

**Figure 1:**
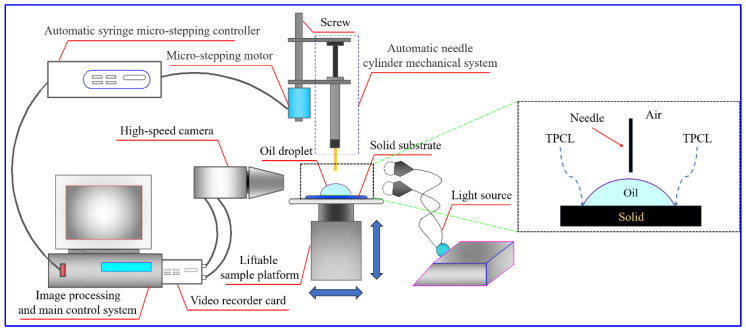
Schematic of the experimental setup for studying the spreading behavior of an oil droplet on the Teflon in air [42].

**Figure 2:**
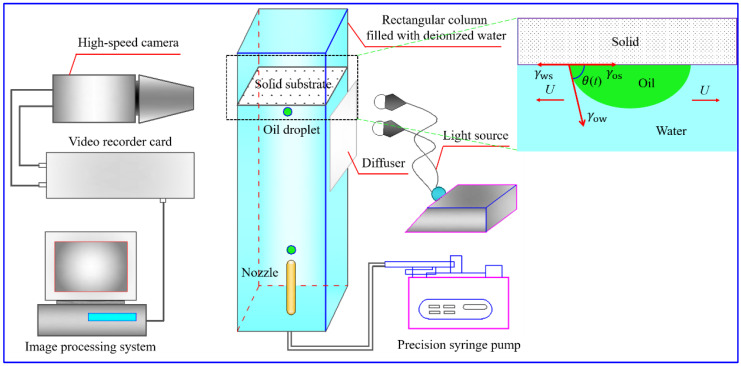
Schematic of the experimental device for studying the spreading behavior of an oil droplet on the Teflon in water [43].

The schematics of the experimental setup for investigating the spreading behavior of an oil droplet on Teflon in air and deionized water are displayed in Figure 1 and Figure 2, respectively. Detailed information on the experimental device structure, experimental process and the processing procedure of recorded images can be found in our previous study [42,43]. Although the measurement process can be found in the literature, some important information also needs to be emphasized here for the oil droplet spreading experiments that occur in different media. For the oil droplet spreading experiment conducted in the air, in order to minimize the influence of the initial kinetic energy of oil droplets on the spreading behavior, the distance between the oil droplet and the Teflon must be adjusted to ensure that the impact velocity of the oil droplet on Teflon surface is less than 0.1 mm/s. The impact velocity is usually determined by calculating the falling height in the first 10 images before the oil droplet touches the surface. Once the oil droplet comes into contact with the surface of Teflon, the spreading process of the oil droplet starts immediately and is recorded by the high-speed camera at a frequency of 1000 fps. Similarly, for the oil droplet spreading experiment performed in the deionized water, the impact velocity of an oil droplet on the Teflon surface is about 22.5 mm/s, and the oil droplet spreading process is also recorded at a frequency of 1000 fps. In addition, the main physical parameters of oleic acid and deionized water used are shown in Table 1. All measurements were conducted at room temperature (around 25 °C), and relative humidity RH = 40 ± 5%.

## 3. Results and Discussion

**Figure 3:**
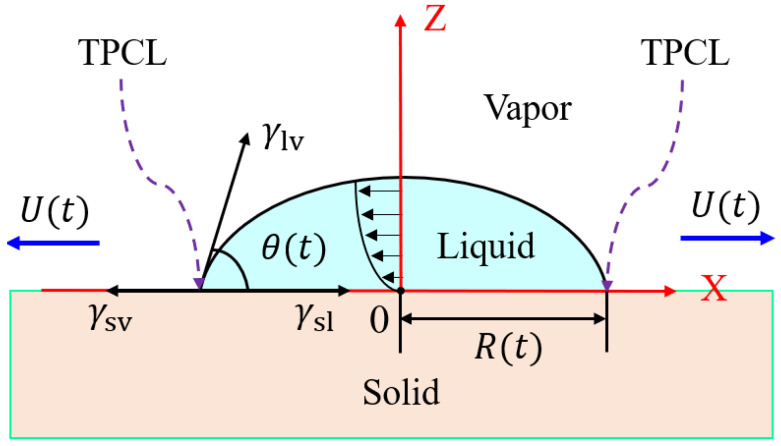
Typical schematic of the spreading of a droplet on a solid surface in the vapor medium, where R(t) is the instantaneous spreading radius, θ(t) is the dynamic contact angle, and U(t) is the velocity of three-phase contact line (TPCL) at t, $\gamma_{\mathrm{sv}}$, $\gamma_{\mathrm{sl}}$ and $\gamma_{\mathrm{lv}}$ are the solid–vapor, solid–liquid, and liquid–vapor interfacial tensions, respectively [44].

The spreading of droplets on solid surfaces is the process of the evolution of droplets from a non-equilibrium configuration to an equilibrium configuration, whose typical schematic diagram is shown in Figure 3. During droplet spreading, the driving force of TPCL motion is compensated by the rate of the dissipation function [44], and the spreading driving force is derived from the loss of surface free energy of the droplet caused by an increase in the instantaneous spreading radius or a decrease in the dynamic contact angle, whereas the dissipation function includes viscous dissipation due to bulk viscous flow, dissipation due to solid–liquid friction near the TPCL and dissipation occurring in the precursor film [45]. Generally, the droplet spreading dynamics are characterized by studying the temporal evolution of the instantaneous spreading radius (R(t)) and dynamic contact angle (θ(t)), which lead to the discovery of these simple scaling laws such as $R\left(t\right)\sim t^{\alpha }$ and $\theta \left(t\right)\sim t^{-3\alpha }$, where the spreading exponent α is usually obtained by fitting the experimental data, which can provide the potential mechanisms governing the droplet dynamic behavior at different spreading stages [46]. It is noteworthy that these simple scaling laws can well describe the spreading dynamics of the fully wetted case, but their application to the partially wetted case has limitations. For the partially wetted case, the temporal evolution of R(t) and θ(t) during droplet spreading still lacks a universal relationship. To attempt to address this issue, a partially wetted experimental system was chosen. In this study, whether in air or in deionized water media, the spreading of oleic acid droplets (oil droplets) on Teflon substrates exhibited partial wetting characteristics (i.e., quasi-equilibrium contact angles much larger than 0°). What is more, to reveal the differences in the spreading behavior of oil droplets on solid surfaces in different media environments and to gain a deeper understanding of the spreading mechanism of oil droplets, we comparably studied the spreading behavior of oil droplets on Teflon in air and in deionized water from the perspective of empirical relations and energy conservation, and the classical HD and MKT wetting models were applied to quantitatively describe the oil droplet spreading dynamics.

### 3.1. Differences in the Spreading Behavior of Oil Droplets on Teflon in Air and Deionized Water

**Figure 4:**
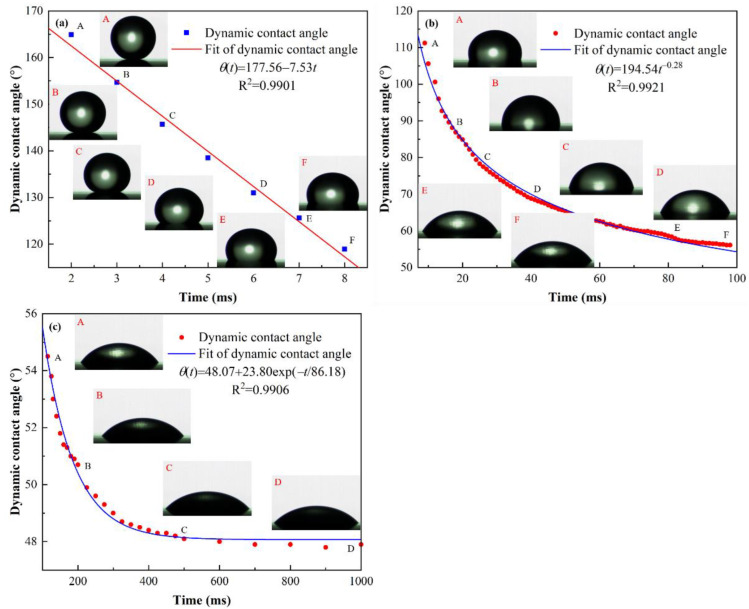
Evolution of dynamic contact angle with time at different time scales during the oil droplet spreading on the Teflon in air. (**a**) *t* < 10 ms, (**b**) 10 ms ≤ *t* < 100 ms and (**c**) 100 ms ≤ *t* ≤ 1000 ms. Note that the scattered dots represent the experimental data, and the solid lines represent the fitting results. The insets correspond to the profiles of an oil droplet at specific moments.

Due to the different formation environment of oil droplets (one is air and the other is deionized water), the size of the oil droplets is slightly different. To make the spreading behavior of oil droplets in the two media comparable, we only comparatively analyzed the dynamics behavior of dynamic contact angle during the oil droplet spreading in air and deionized water from the perspective of empirical relations. Since the spreading of oleic acid droplets on Teflon was a partial wetting process, it could not be perfectly described by the existing scaling law models. Therefore, we empirically divided the entire spreading process of oil droplets for the oil–air–Teflon system into three spreading stages and used linear and nonlinear regression fitting to describe the spreading behavior of oil droplets on Teflon substrates in the air. Figure 4 illustrates the evolution of dynamic contact angle with time at different time scales during the spreading of an oil droplet on the Teflon in air. It can be seen that at *t* < 10 ms, the dynamic contact angle decreased linearly with time, which followed $\theta \left(t\right)=\theta_{0}+kt$; at 10 ms ≤ *t* < 100 ms, the dynamic contact angle decreased exponentially with time, which satisfied $\theta \left(t\right)=bt^{-3\alpha }$; and at 100 ms ≤ *t* ≤ 1000 ms, the decrease of dynamic contact time with time obeyed $\theta \left(t\right)=\theta_{\mathrm{eq}}+a\times \exp\left(-t/T\right)$, where $\theta_{0}$ is the initial contact angle, $\theta_{\mathrm{eq}}$ is the equilibrium contact angle, and T is a tunable parameter related to the physicochemical properties and geometry of the spreading system, which can be used to characterize the time scale required for different spreading systems to reach the equilibrium state. In general, the larger the value of T, the longer it takes to reach the equilibrium state of spreading, and vice versa. These indicated that the early spreading stage, *t* < 10 ms, of oil droplets was quite short, which was dominated by the inertial force [26,43,47,48]. In the intermediate stage of spreading (10 ms ≤ *t* < 100 ms), the temporal evolution of dynamic contact angle could be well described by a simple scaling law of $\theta \left(t\right)\sim t^{-3\alpha }$, and the spreading exponent (−0.28) was close to −0.3 (α=0.1) obtained based on the HD model, which suggested that the spreading of the oleic acid droplet on the Teflon in air during the intermediate spreading process was dominated by the viscous dissipation due to viscous flow in the core of the droplet [27,30]. Furthermore, as oleic acid is a shear-thinning fluid (a non-Newtonian fluid with the rheological power exponent < 1), the spreading exponent α < 0.1 is reasonable [23,41]. In contrast, during the late spreading process (100 ms ≤ *t* ≤ 1000 ms), the simple scaling law, $\theta \left(t\right)\sim t^{-3\alpha }$, could not reasonably describe the temporal evolution of the dynamic contact angle, but the empirical relation, $\theta \left(t\right)=\theta_{\mathrm{eq}}+a\times \exp\left(-t/T\right)$, could fit the experimental data well. This may be because the late spreading dynamics behavior of oil droplets was controlled by a combination of relatively weak bulk viscous dissipation and significant solid–liquid frictional dissipation near the TPCL. In summary, the whole spreading process of oil droplets on the Teflon in air medium could be separated into three stages: the early linear fast spreading stage following $\theta \left(t\right)=\theta_{0}+kt$, the intermediate exponential slow spreading stage obeying $\theta \left(t\right)=bt^{-3\alpha }$, and the late spreading stage described by $\theta \left(t\right)=\theta_{\mathrm{eq}}+a\times \exp\left(-t/T\right)$.

**Figure 5:**
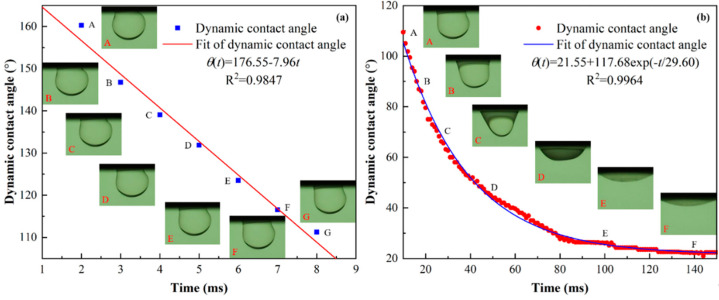
Variation of dynamic contact angle with time at different time scales during the oil droplet spreading on the Teflon in deionized water. (**a**) *t* < 10 ms and (**b**) 10 ms ≤ *t* ≤ 150 ms. Note that the scattered dots represent the experimental data, and the solid lines represent the fitting results. The insets correspond to the profiles of an oil droplet at specific moments.

Similar to the oil–air–Teflon case, according to the dynamics characteristics of oil droplet spreading in the oil–water–Teflon system, we roughly divide the spreading process of oil droplets in deionized water on the Teflon into two stages. Figure 5 displays the variation of dynamic contact angle with time at different time scales during the oil droplet spreading on the Teflon in deionized water. It is clear that at the early spreading stage (*t* < 10 ms), a rapid decrease in the dynamic contact angle was observed, and its temporal evolution followed $\theta \left(t\right)=\theta_{0}+kt$, which could be attributed to the inertial force induced by the kinetic energy of the oil droplet [42]. In contrast, during the late spreading process (10 ms ≤ *t* ≤ 150 ms), the decrease rate of dynamic contact angle gradually became slow, and finally the dynamic contact angle reached a minimum value and remained constant, indicating that the oil droplet spreading reached the equilibrium state. Moreover, at this stage, the variation of dynamic contact angle with time could be well described by $\theta \left(t\right)=\theta_{\mathrm{eq}}+a\times \exp\left(-t/T\right)$, which could be the result of a combination of viscous dissipation of the oil droplet boundary layer and dissipation caused by the surrounding water [40]. In short, the entire spreading process of oil droplets on the Teflon in deionized water could be divided into two stages: the early linear fast-spreading stage following $\theta \left(t\right)=\theta_{0}+kt$, and the late slow-spreading stage described by $\theta \left(t\right)=\theta_{\mathrm{eq}}+a\times \exp\left(-t/T\right)$.

To sum up, it could be easily found that compared with the oil–air–Teflon spreading system, the intermediate exponential spreading stage disappeared in the oil–water–Teflon system, which was the most significant difference in the spreading behavior of oil droplets in the two media environments. To clarify the reason for the absence of the intermediate exponential spreading stage in the oil–water–Teflon system, we theoretically analyzed the spreading process of oil droplets on Teflon substrates in different media environments from the perspective of energy conservation. Despite the different media environments in which oil droplet spreading occurs, there are many similarities in the spreading behavior of oil droplets on solid surfaces. Whether in air or in deionized water media, the total energy of oil droplets includes initial kinetic energy and initial surface energy before contact with the solid surface. Note that in this study, the radius of the oil droplets formed in air and deionized water is much smaller than the capillary length, so the effect of surface tension is dominant compared with the effect of gravity. Therefore, the shape of oil droplets can be considered to be spherical, and in the air medium, the initial kinetic energy (*E*_k,0_) and initial surface energy (*E*_s,0_) of the spherical oil droplet can be expressed as:

(1) $$E_{k,0}=\frac{2}{3}\rho \pi R_{0}^{3}U^{2}$$

(2) $$E_{s,0}=4\pi R_{0}^{2}\sigma_{\mathrm{ov}}$$

where $R_{0}$ is the initial radius of oil droplets, ρ is the density of the oil, U is the impact velocity of oil droplets, and $\sigma_{\mathrm{ov}}$ is the oil–vapor interfacial tension. After contact with the solid surface, the initial kinetic energy drives the oil droplet to spread, and the remaining kinetic energy and interfacial surface energy of the oil droplet are $E_{k,1}$ and $E_{s,1}$, respectively. During oil droplet spreading, its initial kinetic energy is dissipated and converted into interfacial surface energy (*E*_s,1_) and dissipation energy (W) [49]. Since the surface energy and surface roughness of Teflon are very low, the energy dissipation caused by solid–liquid friction during oil droplet spreading can be neglected [50], but the viscous energy dissipation must be considered. Therefore, according to the energy conservation principle, we can obtain that

(3) $$E_{k,0}+E_{s,0}=E_{k,1}+E_{s,1}+W$$

Once in contact with Teflon, the oil droplet will continue to spread on the Teflon surface until the viscous force, surface tension and inertia force are balanced. When the oil droplet spreading reaches the equilibrium state, and then $\;E_{k,1}=0$. Because Teflon is lipophilic, the equilibrium shape of oleic acid droplets on the Teflon surface is a spherical cap and the oil–vapor contact area can be given by [39]

(4) $$S_{\mathrm{ov}}=\frac{2\pi R_{\max}^{2}}{1+\sin\theta_{e}}$$

where $\theta_{e}$ is the equilibrium contact angle, $R_{\max}$ is the spreading radius at equilibrium state.

Therefore, the surface energy of the oil–Teflon system in air can be expressed as [40]

(5) $$E_{s,1}=\frac{2\pi R_{\max}^{2}}{1+\sin\theta_{e}}\sigma_{\mathrm{ov}}+\pi R_{\max}^{2}\left(\sigma_{\mathrm{os}}-\sigma_{\mathrm{vs}}\right)$$

where $\sigma_{\mathrm{os}}$ is the oil–solid interfacial tension in air; $\sigma_{\mathrm{vs}}$ is the vapor–solid interfacial tension.

According to the modified Young’s Equation

(6) $$\sigma_{\mathrm{vs}}=\sigma_{\mathrm{ov}}\cos\theta_{e}+\sigma_{\mathrm{os}}$$

Then Equation (5) can be changed to

(7) $$E_{s,1}=\pi R_{\max}^{2}\sigma_{\mathrm{ov}}\left(\frac{2}{1+\sin\;\theta_{e}}-\cos\theta_{e}\right)$$

Note that because the viscous dissipated energy (*E*_diss_) cannot be calculated directly, it is often estimated using the work done by the viscous friction force [20]. According to Chandra and Avedisian’s expressions [51], the dissipation function ($\psi_{o}$) and dissipation energy (*E*_diss_) can be expressed as:

(8) $$\psi_{o}\approx \mu_{o}{\left(\frac{U}{h}\right)}^{2}$$

(9) $$E_{\mathrm{diss}}=W\approx \psi_{o}V_{o}\tau_{\max}$$

(10) $$\tau_{\max}=\frac{16}{3}\left(\frac{R_{0}}{U}\right)$$

where $V_{o}$, $\mu_{o}$,$\;\tau_{\max}$, and h are the volume of the oil droplet, the viscosity of the oil, the maximum spreading time and the height of oil droplet at maximum spreading state, respectively. Since the distance between the oil droplet and the surface of Teflon is rather short, the impact velocity of the oil droplet is very low, moreover, the oil droplets at the maximum spreading state can be regarded as a spherical cap; therefore, the height of the oil droplet (h) can be given by [39]

(11) $$h=\frac{R_{\max}}{\sin\;\theta_{e}}\left(1-\cos\theta_{e}\right)$$

Substituting Equations (8), (10) and (11) into Equation (9), the total dissipation energy of the spreading process of an oil droplet on the Teflon surface in air is

(12) $$E_{\mathrm{diss}}=W\approx \frac{16\pi R_{0}UR_{\max}\;\mu_{o}\left(3\sin\theta_{e}-1+\cos\theta_{e}\right)}{9\sin\theta_{e}}$$

Due to the similarity in the spreading behavior of oil droplets on solid surfaces in different media, the theoretical analysis of the oil droplet spreading process in the oil–water–Teflon system is basically the same as that in the oil–air–Teflon system, but the most notable difference is energy dissipation. When an oil droplet spreads in the water medium, the dissipated energy includes not only the viscous dissipation of the boundary layer of oil droplet, but also that of the surrounding water which is different from the oil droplet spreading in air [39,40]. Similarly, the viscous dissipation energy (*E*_diss_) can also be estimated using the work done by the viscous friction force, and the dissipation functions of the oil droplet and the surrounding water ($\psi_{o}$ and $\psi_{w}$) and total dissipation energy (*E*_diss_) can be expressed as [51]

(13) $$\psi_{o}\approx \mu_{o}{\left(\frac{U}{h}\right)}^{2}$$

(14) $$\psi_{w}\approx \mu_{w}{\left(\frac{U}{h}\right)}^{2}$$

(15) $$E_{\mathrm{diss}}=W\approx \left(\psi_{o}V_{o}+\psi_{w}V_{w}\right)\tau_{\max}$$

where $V_{o}$, $V_{w}$ are the volume of oil droplet and surrounding water, respectively; $\mu_{o}$ and $\mu_{w}$ are the viscosity of oil and water, respectively.

Due to the action of the surrounding fluid and the low impact velocity of the oil droplet, at the maximum spreading state, the height of the oil droplet (h) for the spherical cap can also be expressed by Equation (11).

Substituting Equations (10), (11), (13) and (14) into Equation (15), the total dissipation energy of spreading process of oil droplet on the Teflon surface in deionized water is

(16) $$E_{\mathrm{diss}}=W\approx \frac{16\pi R_{0}UR_{\max}\left(\mu_{o}+\mu_{w}\right)\left(3\sin\theta_{e}-1+\cos\;\theta_{e}\right)}{9\sin\theta_{e}}$$

Based on the above theoretical analysis, we can know that whether in air or in water media, during the oil droplet spreading on solid surfaces, the dynamic behavior of oil droplets and the energy conversion form of the system are basically the same. Furthermore, the total dissipation energy of the system depends mainly on the size of the oil droplets, surface wettability, impact velocity and fluid viscosity. These make the spreading behavior of oil droplets in different media environments have numerous similarities. For example, the early rapid spreading stage follows $\theta \left(t\right)=\theta_{0}+kt$ and the late spreading stage satisfies $\theta \left(t\right)=\theta_{\mathrm{eq}}+a\times \exp\left(-t/T\right)$, as shown in Figure 4 and Figure 5. However, the most remarkable difference of the oil droplet spreading in different media environments is found to be the difference in the dissipation energy of the system. According to Equations (12) and (16), the total dissipation energy of the oil-water-Teflon system includes not only the viscous dissipation energy of the oil droplet boundary layer, but also that of the surrounding water which is not included in the total dissipation energy of the oil-air-Teflon system. Therefore, compared with the oil-air-Teflon system, the absence of the intermediate exponential spreading stage in the oil-water-Teflon system can be attributed to the difference in the dissipation energy of the system; more precisely, the viscous dissipation energy caused by the water surrounding the oil droplets.

### 3.2. Analysis of Oil Droplet Spreading Dynamics Based on HD and MKT Models

HD and MKT models are two classical models for describing droplet spreading dynamics, which reveal the spreading mechanism of droplets on solid surfaces by studying the relationship between TPCL velocity and dynamic contact angle. Their difference lies in the source of the dissipated energy of the system during droplet spreading. The HD model assumes that the energy dissipation of the system is the viscous dissipation caused by the viscous flow in the bulk of the droplet, and the bulk viscous friction is the main resistance force for the TPCL movement, and the relationship between the TPCL velocity (U) and the advancing dynamic contact angle ($\theta_{A}\left(t\right)$) can be expressed as [27,28]

(17) $$\theta_{A}{\left(t\right)}^{3}=\theta_{e}^{3}+\frac{9\eta }{\gamma }\mathrm{In}\left(\frac{L}{L_{s}}\right)U$$

where η is the viscosity of the liquid droplet, γ is the interfacial tension of two immiscible fluids, L is a characteristic length parameter defined as the capillary length ($L=\sqrt{\gamma /\rho g}$, ρ is the density of the liquid droplet, g is the gravitational acceleration), and the slip length $L_{s}$ is usually determined by fitting experimental data, and it should be in the order of the molecular dimension because its value reflects the scale of the region where the no-slip boundary condition of classical continuum theory does not hold [30]. The linear HD model of Equation (17) indicates that the droplet spreading velocity (U) is proportional to the cube of advancing dynamic contact angle ($\theta_{A}{\left(t\right)}^{3}$). The MKT model believes that during droplet spreading, the energy dissipation of the system is the dissipation due to the solid–liquid friction effect in the vicinity of TPCL, and the macroscopic behavior of TPCL is determined by the overall statistics of the individual molecular displacements within the TPCL region [29], and the relationship between the TPCL velocity (U) and the advancing dynamic contact angle ($\theta_{A}\left(t\right)$) can be given by

(18) $$U=2K^{0}\lambda \;\sin h\left[\frac{\gamma \left(\cos\theta_{e}-\cos\theta_{A}\left(t\right)\right)}{2nk_{B}T}\right]$$

where $K^{0}$ is the quasi-equilibrium frequency of molecular replacements, λ is the characteristic length of individual molecular displacement, $k_{B}$ is the Boltzmann constant, T is the absolute temperature, and n is the adsorption site density, which is approximately equal to $1/\lambda^{2}$ when the adsorption sites on the solid surface are uniformly distributed. Since sinh in Equation (18) is small when $\theta_{A}\left(t\right)$ is approximately equal to $\theta_{e}$ [52], and then Equation (18) can be simplified to the linear form

(19) $$U=\xi \gamma \cos\theta_{e}-\xi \gamma \cos\theta_{A}\left(t\right)$$

where the parameter $\xi =K^{0}\lambda^{3}/k_{B}T$ is defined as the TPCL friction coefficient, which is often used to estimate energy dissipation in the TPCL region, and is usually treated as a constant for a simple dynamic process. Similarly, the linear MKT model of Equation (19) suggests that the TPCL velocity (U) is proportional to the cosine functions of advancing dynamic contact angle ($\cos\theta_{A}\left(t\right)$). As mentioned above, although the two models have been confirmed in some specific experimental systems and can explain the dynamics behavior of TPCL, in many cases, they do not alone describe the experimental data for the entire TPCL velocity range. In order to better describe the spreading dynamics of oil droplets and further understand the spreading mechanism of oil droplets on solid surfaces in different media environments, the classical HD and MKT models were applied to quantitatively analyze the relationship between TPCL velocity (U) and advancing dynamic contact angle ($\theta_{A}\left(t\right)$) during the spreading of oil droplets on the Teflon surface in air and in water media by the plots of $\theta_{A}{\left(t\right)}^{3}\;–\;U$ and $\cos\theta_{A}\left(t\right)\;–\;U$, respectively.

**Figure 6:**
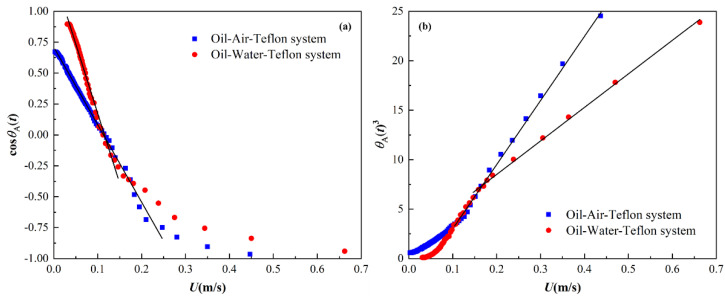
Relationship between TPCL velocity (U) and advancing dynamic contact angle ($\theta_{A}\left(t\right)$) during the spreading of oil droplets on the Teflon surface in different media environments under the MKT and HD model frameworks, respectively. The scattered dots represent the experimental data, and the solid lines represent the fitting results of MKT model (**a**) and HD model (**b**), respectively.

Figure 6a,b show the relationship between TPCL velocity (U) and advancing dynamic contact angle ($\theta_{A}\left(t\right)$) during the spreading of oil droplets on the Teflon surface in different media environments under the MKT and HD model frameworks, respectively. It should be mentioned that whether in air or in deionized water, U gradually decreased until it became zero at the equilibrium state as oil droplet spreading progressed (i.e, $\theta_{A}\left(t\right)$ decreased). It could be observed from Figure 6a that for the oil–air–Teflon and oil–water–Teflon spreading systems, all data points in the low-TPCL-velocity range (U<0.15 m/s) satisfied the linear trend of Equation (19), indicating that the MKT model could well describe the dynamics behavior of oil droplets at the late spreading stage. However, a significant deviation between the measured and predicted cosθ(t) was observed at the early spreading stage and the intermediate spreading stage (large U values, i.e., U>0.15 m/s), which seemed to imply that the spreading dynamics of oil droplets at the early and intermediate spreading stages was beyond the application of MKT model [34,53]. Interestingly, the HD model could perfectly describe the dynamics behavior of oil droplets at the early and intermediate spreading stages (U>0.15 m/s), as shown in Figure 6b, but it could not well describe the late spreading dynamics of oil droplets (U<0.15 m/s). In conclusion, the MKT model could reasonably describe the oil droplet spreading dynamics in the low-TPCL-velocity range, while the HD model was more suitable for describing that in the medium- to high-TPCL-velocity range.

## 4. Conclusions

In this work, we adopted the high-speed dynamic visualization technology to investigate comparatively the spreading behavior of millimeter-sized oleic acid droplets (oil droplets) on the smooth hydrophobic Teflon substrate in air and water media, and analyzed their differences from the perspective of empirical relations and energy conservation. Meanwhile, to better describe the spreading dynamics of oil droplets and further understand the spreading mechanism of oil droplets on the Teflon in different media environments, the classical HD and MKT models were applied to quantitatively analyze the relationship between TPCL velocity (U) and advancing dynamic contact angle ($\theta_{A}\left(t\right)$). On the one hand, the whole spreading process of oil droplets on Teflon in the air medium could be divided into three stages: the early linear fast-spreading stage, the intermediate exponential slow-spreading stage and the late spreading stage. However, the intermediate exponential spreading stage disappeared in the oil–water–Teflon system compared with the oil–air–Teflon spreading system, which was mainly attributed to the difference in the dissipated energy of the system, derived from the theoretical analysis based on the principle of energy conservation, because the dissipation energy in the oil–water–solid system included not only the viscous dissipation energy of the boundary layer of oil droplet, but also that of the surrounding water which was not included in the dissipation energy of the oil–air–Teflon system. On the other hand, whether it was the oil–air–Teflon spreading system or oil–water–Teflon spreading system, the MKT model could well describe the dynamics behavior of oil droplets at the late spreading stage (corresponding to the low-TPCL-velocity range), whereas the HD model was more suitable for describing the oil droplet spreading dynamics at the intermediate and early spreading stages (corresponding to the medium- to high-TPCL-velocity range). These findings can provide guidance for revealing the dynamic wetting mechanism of oil droplets on solid surfaces in different media.

## Figures and Tables

**Table 1:** Main physical parameters of oleic acid and deionized water (25 °C).

Physical Parameters	Unit	Oleic Acid	Deionized Water
Density	kg/cm^3^	893.5	997.0
Viscosity	mPa s	27.64	0.897
Air–liquid interfacial tension	mN/m	33.80	72.80
Oil–water interfacial tension	mN/m	16.35	-
Droplet diameter (in water)	mm	3.14	-
Droplet diameter (in air)	mm	3.05	-

## Data Availability

Not applicable.
